# Isolation and identification of microorganisms from public and semi-public multi-user video gamepads with respect to hygiene and disinfection measures

**DOI:** 10.3934/publichealth.2026028

**Published:** 2026-04-27

**Authors:** Abdullah Alfarhan, Muhammad Poyil, Saad Alshahrani, Elmutasim Ibnouf, Maged Abdel-Kader

**Affiliations:** 1 Department of Pharmaceutics, College of Pharmacy, Prince Sattam Bin Abdulaziz University, P.O. Box 173, Al-Kharj 16273, Saudi Arabia; 2 Department of Basic Medical Sciences, College of Medicine, Prince Sattam Bin Abdulaziz University, P.O. Box 173, Al-Kharj 11942, Saudi Arabia; 3 Department of Medical Microbiology, Faculty of Medical Laboratory Sciences, Omdurman Islamic University, Sudan; 4 Department of Pharmacognosy, College of Pharmacy, Prince Sattam Bin Abdulaziz University, P.O. Box 173, Al-Kharj 11942, Saudi Arabia

**Keywords:** video gamepads, microorganisms, disinfection, public, semi-public

## Abstract

The purpose of the current study was to evaluate the possible role of multi-user video gamepads in spreading microbial populations among users and understand how to reduce this risk. The study design involved 10 public locations in Al-Kharj city and 10 semi-public locations inside the campus of Prince Sattam Bin Abdulaziz University, Al-Kharj, Saudi Arabia. Air samples were collected from all selected places once during the study using a passive air sampling technique. Semi-public places with a good aeration system and a regular cleaning routine showed much less airborne organisms than public places with less care. Samples from the multi-user video gamepads were taken from each gamepad three times during a period of two weeks. Each time, one sample was taken before any intervention, and another sample was collected after disinfection with commercial alcohol swab pads containing 70% isopropyl alcohol. All samples were examined for the number of viable organisms. The mean microbial count ranged from 98 to 270 CFU in public locations and from 18 to 34 CFU in semi-public locations. Samples collected from semi-public places showed 3 bacterial genera and were free from fungi, while samples from public places showed 9 bacterial and 4 fungal genera. After disinfection of the multi-user video gamepads in the public places, the number of colonies reached a maximum of 8 CFU (approximately 97% reduction), while the semi-public places expressed a 91% reduction in the number of organisms. The results obtained demonstrate the importance of following hygiene measures in multi-user objects to reduce the risk of infection transmission.

## 1. Introduction

Microorganisms are extremely small organisms that can only be seen through a microscope [1]; they have both positive and negative impacts on humans. Bacteria, fungi, and viruses are examples of these organisms [2]. Microorganisms exist everywhere and can live in new environments and dramatically increase their numbers in a short period of time. These properties are fundamental to their existence in the depths of the Earth's crust and on the surfaces of soil, wastewater, and hot springs, as well as the living bodies of plants, animals, and organic matter [3]. Many Gram-positive bacteria, such as *Staphylococcus aureus, Enterococcus* spp., and *Streptococcus pyogenes*, and Gram-negative bacteria, like *Escherichia coli, Acinetobacter* spp., *Pseudomonas aeruginosa, Klebsiella* spp., and *Shigella* spp., can survive on solid surfaces for months [4]. Most pathogenic organisms have a variable survival rate, with *Mycobacteria* and *Clostridium difficile* living for months, and *Vibrio cholera, Haemophilus influenza*, and *Bordetella pertussis* living only for days [1]. Bacteria such as *Salmonella* and *Escherichia coli* have been linked to the transfer of bacteria from the hands to raw, processed, and cooked foods [5].

There are several scientific investigations suggesting the role of cross-contamination in the transmission of many nosocomial infections. These infections are caused by various multi-drug-resistant (MDR) strains of bacteria, viruses, and fungi [6],[7]. Drug-resistant bacterial species include methicillin-resistant *Staphylococcus aureus* (MRSA), vancomycin-resistant *Enterococci* (VRE), *Clostridium difficile*, *Pseudomonas aeruginosa*, and *Acinetobacter baumannii*, among others [6],[8].

Microorganisms have resulted in many pandemics and epidemics throughout human history, leading to the loss of millions of lives caused by bacteria such as plague and cholera or viruses such as flu, Middle East respiratory syndrome coronavirus (MERS-CoV), and severe acute respiratory syndrome coronavirus (SARS-CoV) [6]. The world is still fighting the disastrous pandemic caused by the coronavirus disease in 2019, known as COVID–19. The first step in controlling and preventing such infections is to understand the mechanisms of transmission of these pathogens among people [9]. Five main modes of transmission of bacterial infections are well documented and can be summarized as vectors biting insects or animals, and contact, airborne, droplet, and vehicular transmission [10]. Bioaerosols significantly impact human life, potentially causing infections, allergies, and toxic effects. Assessing microbial air quality is a crucial aspect of risk management: it enables confirmation of biological agents, identifies critical conditions, and validates implemented preventive measures. Air sampling also serves as a valuable resource for scientific research, quality control, and educational uses. Microbial air sampling methods can be divided into two categories: passive and active. Viruses mainly invade human bodies via the host epithelium, which represents the first natural barrier between the body and the outside environment [10]. In some cases, infections can occur through transplantation of a virally infected organ [11]. Contaminated food, drinks, and water play an important role in spreading infections, especially in developing countries [10].

One critical mode of transmission of microbial pathogens is the hand-to-hand contact or contact with contaminated fomites [10],[12]. Some microbes can survive for hours to weeks on nonporous surfaces and can be infectious at very low doses [13]. In our modern life, contaminated objects such as countertops, telephone hand pieces, door handles, and everyday household objects may result in the passage of microbes to the mouth after handling [13]. The ability of these pathogens to survive for more than 24 hours increases their chances of spreading infections into other places [14].

Scientific investigations have shown the persistence of pathogenic microorganisms on various surfaces of frequent public contact by a diverse population. A study in a casino environment identified the presence of bacterial and fungal pathogens, including *E. coli*, at a significant level, on casino chips [15]. Other surfaces, such as computer keyboards and mobile phones, have also shown the presence of bacterial pathogens like *Staphylococcus aureus*, coagulase-negative *staphylococci, Clostridium perfringens, Bacillus* spp., *Streptococcus* spp., Enterobacteriaceae family members, and fungal isolates [16],[17]. Gamepads and computer keyboards used by digital gamers with different hygienic habits and domestic and public stations are of great importance for infection transmission. Millions of young people around the globe are addicted to video games, so much so that the World Health Organization (WHO) considered *gaming disorder* as a new diagnostic category in the WHO ICD–11 (International Classification of Diseases, 11th revision) [18].

Virtually, no abiotic surface with human contact is free from infectious microbial agents. Door handles in public transportation facilities are also known to be the habitat of drug-resistant strains of bacterial pathogens, including *Staphylococcus aureus, Streptococcus faecalis, Staphylococcus epidermidis, Klebsiella* spp., *Escherichia coli, Pseudomonas aeruginosa, Proteus* spp., *Enterobacter* spp., *Bacillus* spp., and *Micrococcus* spp. [19].

A recent study assessed the risk of infectious disease transmission in public washrooms, including COVID–19, and indicated that many practices could result in widespread bacterial and/or viral contamination. These practices include uncovered rubbish bins, blocked drains, open-lid toilet flushing, ineffective handwashing and/or hand drying, and infrequent surface cleaning [20]. Another study evaluated the effect of insufficient ventilation in public toilets on increasing the risks for cross-infection. The study proved that bacterial colony-forming units (CFUs) in public toilets with poor ventilation may reach up to 5 times the number of CFUs outside of the toilet [21]. Aerosols containing either bacteria or fungal spores can result in the spread of respiratory disorders and hypersensitivity reactions. Nearly 10% of all allergies are caused by fungi [22],[23]. The microbial load on public facilities is affected by various factors, including the nature and awareness of users and society, the season, and the facility's vicinity. In a study on game carcasses, Paulsen et al. (2004) [24] showed that there is a significant effect of seasonal changes on the microbial population. Another research conducted in rural and urban primary health care centers concluded that the socio-economic status of the population can also affect the microbial population of public-contacting surfaces [25].

At the same time, disinfection has been proven to be the most effective way to reduce or eliminate these cross-contaminating infectious agents. Various studies have evaluated the degree of effectiveness of the same type of disinfecting agent at different levels. A study by Koscova et al. [16] found that the disinfection of computer keyboards and mobile phones could drastically reduce the microbial population, with an effectiveness between 36.8% and 100%. Another investigation showed that artificial contamination by rotavirus, noroviruses, influenza virus, parechovirus, poliovirus, adenovirus, *Salmonella enterica*, and *Staphylococcus aureus* on stainless steel surfaces was reduced dramatically after a single-wipe liquid soap chlorine solution [26]. Frequent public handling surfaces, including tap handles, contaminated with *Staphylococcus aureus* and *Escherichia coli*, were also reported to have a mean log10 contamination reduction (LR) [27].

The goal of the present study is to isolate and identify the microorganisms present on domestic and public multi-user video gamepads and assess the role of hygienic and disinfection measures in reducing the risk of infection transmission.

## 2. Materials and methods

### 2.1. Materials and reagents

Glassware, such as McCartney bottles, beakers, conical flasks, measuring cylinders, glass slides, inoculating wire loops, aluminum foil, cotton wool, and swab sticks, was used. Distilled water, ethanol, and stains like crystal violet, safranin, and Gram iodine were also utilized.

### 2.2. Preparation of media

Media (nutrient agar, McConkey agar, and Sabouraud dextrose agar) were prepared following the manufacturer's specifications. Prepared media were packed and sterilized at 121 °C for 15 min in the autoclave. The media was cooled to approximately 45 °C before use.

### 2.3. Sample collection and processing

#### 2.3.1. Sampling locations

The samples were taken from the air and multi-user video gamepads at public and semi-public sites.

#### 2.3.2. Air sample collection and analysis

The passive technique assesses how quickly microorganisms adhere to surfaces; it depends on sedimentation and utilizes settle plates that are exposed to air for a specific duration. Outcomes are represented as CFU/plate/time. The Index of Microbial Air Contamination (IMA) standardized the passive method, which reflects the number of CFU measured on a Petri dish exposed to air following the 1/1/1 protocol (for 1 h, 1 m above the ground, and approximately 1 m from walls and significant obstructions) [28].

Nutrient agar in Petri dishes was exposed to the air in the tested places for 60 min (passive air sampling method) for the ambient air sampling for aerial microorganisms. The dishes were sealed, immediately transferred to the lab, and incubated for 24 h at 37 °C. Then, colonies were examined. Experiments were run in triplicate.

#### 2.3.3. Multi-user video gamepads sample collection

A total of 140 samples were collected, including 20 air samples. From the 10 public and 10 semi-public stations, 120 swabs were collected during the experimental period. Public gaming areas, such as cafés or open-access social gaming spaces, were characterized by continuous daily user flow and unrestricted public access. Users of different ages, educational levels, and social backgrounds were freely allowed to use these facilities. Sampling was conducted between 6:00 PM and 10:00 PM to meet the peak times of public gathering at entertainment venues. It was estimated that approximately 50 people were present in each facility during this period.

Semi-public places, on the other hand, are located in Prince Sattam Bin Abdulaziz University. Stations are located in specified rooms with a restricted number of participants, all being university students. Sampling was conducted between 12:00 PM and 2:00 PM during active gameplay sessions during students' breaks. It was estimated that 20 participants were present during this time. Both settings involved active multiplayer gaming sessions requiring full use of all gamepad buttons, ensuring comparable surface contact during gameplay.

Swab samples were collected using Deltalab Amies liquid Swab (Code 300284.SE) from the multi-user video gamepads at the studied locations twice on the same day. The first-time samples were collected without any intervention; then, video gamepads were disinfected. The disinfection protocol was standardized across all sites using 70% isopropyl alcohol swab pads (Saudi Medical Circle Code: 427–001). Each gamepad, including directional buttons, action buttons, sticks, and side grips, was thoroughly wiped to ensure complete surface coverage for 1 min. The surface was left to air-dry and ensure sufficient contact time. The second swabs were collected immediately after disinfection, which was consistently applied in all locations. Samples were collected from the selected public and semi-public places on three different days, evenly distributed during the study period. The swab sticks were transferred directly to the laboratory within a maximum of 2 h for bacteriological analysis. Experiments were run in triplicate.

### 2.4. Bacterial inoculation

Bacterial inoculation was accomplished through direct streaking of the swab sticks onto the surface of the nutrient agar in Petri dishes. Sterile swabs were used, and care was taken not to contaminate them by touching other surfaces. The transfer to growth media was done quickly. The swab was held comfortably in one hand, and the lid of the Petri dish was lifted with the other hand. A Petri dish cover was used to protect the agar from air contamination; the swab was gently pulled across the surface of the agar in a zigzag pattern. This way, single bacterial cells get isolated by the streak; when the plate is incubated, they form discrete colonies that start from just one bacterium each. The lid was placed on the base of the Petri dish. The swab was disposed of in an appropriate receptacle (e.g., Biohazard bag). The bases of the plates were labeled with the name, date, sample code, and environmental source. Petri dishes were incubated for 24 h at 37 °C before colonies were examined.

### 2.5. Isolation and identification of bacterial isolates

Petri dishes were examined for growth 24 h after incubation; isolated colonies were then sub-cultured on fresh media Petri dishes until pure isolates were observed. Pure cultures of the isolates were then stocked into McCartney bottles. After satisfactory growth, organisms were identified based on their morphological characters, reaction to Gram stain, and biochemical characteristics [1].

### 2.6. Gram staining techniques

A thin smear was created on a grease-free slide by liquefying a small number of microbes from a stocked colony of 18–24-h-old pure culture in a drop of sterile distilled water. The smear was completely dry before being heat-fixed by passing it briefly over a flame. The slide was placed on the staining rack and stained for 30–60 s with crystal violet as a primary stain. Gram's iodine was added (mordant) for 30 s. The smear was then gently washed with tap water. Ethyl alcohol 70% was used for decolonization for 10–30 s before staining with the secondary stain safranin for 30 s. The smear was then washed with tap water and air-dried. The prepared smear was finally investigated with the oil immersion lens under the light microscope. Gram-positive organisms were purple, while Gram-negative organisms were red.

### 2.7. Biochemical characterization of isolates

Tests were carried out to further identify and classify the bacterial isolates. These tests included the coagulase test, catalase test, oxidase test, citrate utilization test, motility test, indole test, urease test, and sugar fermentation test [1],[28].

#### 2.7.1. Coagulase test

Coagulase is an enzyme that aids in the clotting of blood plasma. Gram-positive *Staphylococcus aureus* was subjected to this test. A drop of sterile distilled water was placed on each end of a sterile slide. A test organism colony was emulsified at each location to create thick suspensions. A loop of plasma was gently mixed into one of the suspensions. Within 10 s, the slide was examined for clumping or clotting of the organism. The plasma was not added to the second, control suspension.

#### 2.7.2. Catalase test

This test detects organisms that produce the catalase enzyme, which detoxifies hydrogen peroxide (H_2_O_2_) by breaking it down into water and oxygen gas. A drop of 3% hydrogen peroxide solution was added to the sterile slide containing the organism. A positive outcome was indicated by foaming or bubbles.

#### 2.7.3. Oxidase test

This test is used to identify microorganisms that contain the cytochrome oxidase enzyme (important in the electron transport chain), being commonly used to differentiate between oxidase-negative *Enterobacteriaceae* and oxidase-positive *Pseudomonadaceae*. A few drops of oxidase reagent (Tetramethyl-*p*-phenylenediaminedihydrochloride) were placed on a piece of filter paper. The test organism colony was then smeared on the soaked filter paper. The phenylenediamine in the reagent is oxidized to a deep purple color if the organism produces oxidase. A color change within 10 s indicates a positive outcome.

#### 2.7.4. Citrate utilization test

This test is frequently used to distinguish organisms that can use citrate as a carbon source. In a bijou bottle, Simmon's citrate agar medium was prepared and allowed to set in a slanting position. The test organism was inoculated onto the slant medium using a sterile wire loop and incubated at 37 °C for 48 h before being examined for color change. A bright blue color in the medium indicates a positive citrate test.

#### 2.7.5. Indole test

This test is used to identify microbes capable of converting tryptophan to indole. It is used to identify bacteria in the *Enterobacteriaceae* family. Sterilized tubes inoculated with tryptophan broth (4 mL) were incubated for 24–28 h, and then 0.5 mL of Kovac's reagent was added. The presence or absence of a ring indicates a positive or negative test.

#### 2.7.6. Urease test

This test is used to identify organisms that can hydrolyze urea (bacteria that produce urease) to produce ammonia and carbon dioxide, being mainly used to distinguish urease-positive *Proteases* from other *Enterobacteriaceae*. Organisms that rapidly hydrolyze urea give a strong positive reaction within 1–6 h after incubation. Late-positive organisms give weak positivity on the slant after 6 h of incubation and become stronger with further incubation. If microorganisms are urease-negative, the medium retains a yellowish color. Isolated bacteria were inoculated onto slanted urea agar tubes prepared according to the manufacturer's instructions and incubated at 37 °C for 18–24 h. When the organism produces the urease enzyme, the color changes from light orange to magenta. If the organism does not produce urease, the slope and bottom will remain bright orange (medium retains its original color).

#### 2.7.7. Sugar fermentation test

A carbohydrate fermentation test is used to determine whether bacteria can ferment a particular carbohydrate. Carbohydrate fermentation patterns help distinguish groups or species of bacteria, and this test analyzes the presence of acids and/or gases produced during carbohydrate fermentation. For this purpose, basal media containing a single carbohydrate source, such as glucose, lactose, sucrose, or other carbohydrates, were used. A pH indicator, bromothymol blue (BTB), was also included in the medium. The pH of a drop of the medium was measured to detect acid production. To test the production of gas (hydrogen or carbon dioxide), Durham tubes were immersed in the medium. All members of *Enterobacteriaceae* give positive results.

For fungi, the germ tube, *Candida* chromogenic medium, carbohydrate assimilation, caffeic acid disk, and nitrate assimilation tests were applied [29].

#### 2.7.8. Statistical analysis

Values obtained from three replicates of each experiment are presented as the mean ± standard deviation. Student's t-test was applied, and differences were considered statistically significant at P < 0.05.

## 3. Results

**Figure 1.**
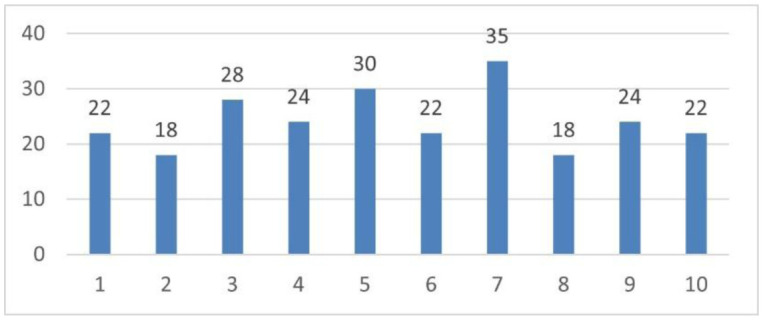
Number of microorganisms (CFU) in air samples from 10 public places.

**Figure 2.**
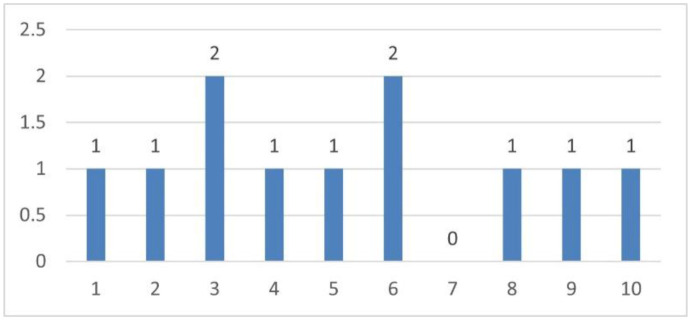
Number of microorganisms (CFU) in air samples from 10 semi-public places.

Twenty air samples were collected from 10 public and 10 semi-public locations and examined for the corresponding microbial counts (Figures 1 and 2). The mean microbial count in the public places ranged from 18 to 36 CFU, and that of semi-public places ranged from 1 to 2 CFU.

**Figure 3.**
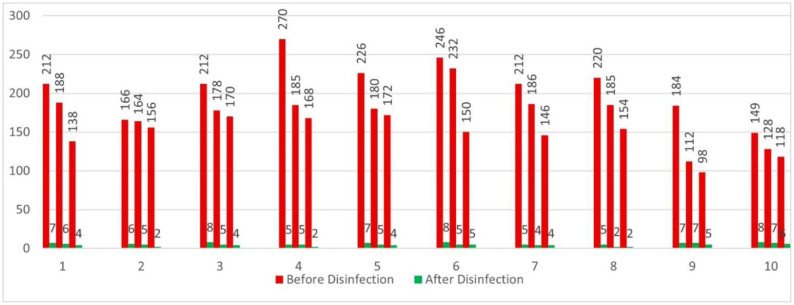
Number of microorganisms (CFU) in multi-user video gamepad samples collected from 10 public places three times in two weeks before and after disinfection.

**Figure 4.**
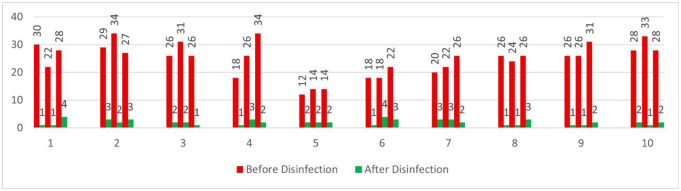
Number of microorganisms (CFU) in multi-user video gamepad samples collected from 10 semi-public places three times in two weeks before and after disinfection.

Samples collected from 10 public locations three times during a period of two weeks from each location showed a mean microbial count ranging from 98 to 270 CFU (Figure 3); those obtained from semi-public locations in the same fashion and time frame had much lower values (18–34 CFU) even before disinfection (Figure 4). After cleaning the multi-user video gamepads using alcohol swab pads containing 70% isopropyl alcohol, the number of detected colonies was dramatically reduced. In public locations, the number of colonies reached a maximum of 8 CFU, with a reduction of approximately 97% in the number of detected organisms. Semi-public places presented only 1 colony, and the maximum number detected was 4 in two cases, for a reduction in the number of organisms of more than 91%.

The type of contaminating microorganisms was also assessed (Table 1). Samples from semi-public locations showed only three bacterial genera, namely *Staphylococcus*, *Bacillus*, and *Lactobacillus* spp. All three were Gram-positive bacteria. On the other hand, nine bacterial genera were detected in samples collected from public locations. Four out of the nine were Gram-negative organisms.

The fungal contaminants found in both public and semi-public places are presented in Table 2.

**Table 1.** Bacterial genera identified in the multi-user video gamepad samples collected from 10 semi-public and 10 public places.

Sample no.	Bacterial genera	Type	Sample location	
			Semi-public	Public
1	*Staphylococcus* spp.	Gram-positive	√	√
2	*Streptococcus* spp.	Gram-positive	X	√
3	*Bacillus* spp.	Gram-positive	√	√
4	*Enterococcus* spp.	Gram-positive	X	√
5	*Micrococcus* spp.	Gram-positive	X	√
6	*Campylobacter* spp.	Gram-negative	X	√
7	*Klebsiella* spp.	Gram-negative	X	√
8	*Salmonella* spp.	Gram-negative	X	√
9	*Escherichia coli*	Gram-negative	X	√
10	*Lactobacillus* spp.	Gram-positive	√	X

**Table 2.** Fungal genera identified in the multi-user video gamepad samples collected from 10 semi-public and 10 public places.

Sample no.	Fungal genera	Sample location	
		Semi-public	Public
1	*Aspergillus* spp.	X	√
2	*Mucor* spp.	X	√
3	*Rhizopus* spp.	X	√
4	*Penicillium* spp.	X	√

## 4. Discussion

Different studies have indicated that solid surfaces in sports and recreational environments can be among the major sources of bacterial contamination. Microbiological analysis of sand and water samples at 10 different recreational beaches in Northeast Ohio found an overall prevalence of *Staphylococcus aureus* in 22.8% of the samples [30]. Another study revealed the presence of *S. aureus*, coagulase-negative *Staphylococcus* species, and *Bacillus* species in the indoor air of primary school classrooms in Gondar city, Northwest Ethiopia [31]. Several different bacterial species, including pathogenic strains, were isolated during a microbiological survey on 300 door handles in 15 secondary schools in Nigeria [32]. The isolates included *S. aureus*, *S. saprophyticus*, *Bacillus mycoides*, *B. subtilis*, *B. megaterium*, *Pseudomonas fluorescens*, *P. aeruginosa*, *Escherichia coli*, *Klebsiella oxytoca*, *Enterobacter* spp., and *Citrobacter freudii*. An investigation carried out by Cheatham et al. (2019) on 259 environmental samples from different rural, suburban, and urban nursing homes in Northeast Ohio, USA, found that the prevalence of *S. aureus* was 28.6% [33]. A study on the doorknobs of washrooms at Daeyang Luke Hospital, Malawi, showed that 41.6% of the samples had been contaminated by various bacterial species, including *S. aureus, E. coli, P. aeruginosa, Citrobacter* spp., and *Klebsiella pneumonia* [34]. Solid mobile phone and computer keyboard surfaces were found to be contaminated with pathogenic bacteria, such as *Staphylococci*, *Bacillus*, and the family Enterobacteriaceae, which were reduced by 36.8%–100% by wiping with disinfectants [35]. Another microbiological survey on the microbial contamination of inanimate surfaces in an ICU, including computer keyboards, at Kashan University hospitals, showed the presence of coagulase-negative staphylococci at 76% [36]. Gerba and Maxwell (2012) evaluated the presence of *E. coli* on the handles and sheets of grocery shopping carts, revealing contamination with bacteria on 51% of the samples [37]. Microbial samples collected from kitchens of houses, restaurants, food processing kiosks, and hotels proved to be contaminated with pathogenic species of bacteria, including *Bacillus* spp., *S. epidermidis*, *S. aureus*, *Shigella* spp., *E. coli*, *Pseudomonas* spp., *Enterobacter* spp., and *Enterococcus* spp. [38]. A study conducted in Jeddah city, Saudi Arabia, on four different solid surfaces commonly encountered by the public, like computer keyboards, computer mice, shopping cart handles, and elevator buttons, showed that 95.5% of the analyzed samples were contaminated with different bacteria [13]. The findings of such studies conducted across the globe show the presence of bacterial species on different objects continuously exposed to social contact, including pathogens.

This study aimed to identify the number and types of microorganisms in air samples and video game samples from various locations. The first group of samples was collected from general consumer products, excluding electronics. Five cafes and five computer centers in Al-Kharj, Saudi Arabia, were selected. Air samples from the ten locations were collected once from each location. On the other hand, video gamepad samples were collected three times from each location on three different days for a period of two weeks. On each day, samples from the gamepads were collected once without any intervention, followed by a second sample after disinfection. Similarly, 10 samples were collected from different locations at Prince Sattam Bin Abdulaziz University (PSAU) activity rooms, located in different colleges. These locations can be described as semi-public places, as they are restricted to PSAU students only. These activity rooms are well aerated and cleaned on a regular basis, following the routine measures applied in all university facilities.

The results of air samples, expressed as the number of colonies observed after incubation of the nutrient agar Petri dishes exposed to air in the selected locations for 60 min (passive air sampling method) (Figures 1 and 2), revealed a significant difference between the public and semi-public places. The semi-public places had mean microbial counts ranging from 1 to 2 CFU; on the other hand, the lowest count in public places was 18 CFU, reaching a maximum of 35 CFU. These results highlight the critical role of aeration and cleaning on a regular basis in reducing the risk of transmission of airborne pathogens [39].

Multi-user video gamepads can be a source of contact pathogen transmission. These gamepads are touched by many users daily; if not periodically cleaned, they can present high risks for users, especially during epidemics or pandemics. Swab samples collected from multi-user video gamepads at the studied locations were transferred without delay to the laboratory and streaked onto the surface of nutrient agar Petri dishes, followed by incubation for 24 or 48 h at 37 °C to allow bacterial and fungal growth, respectively. The comparison between microbial counts of samples collected from the public and semi-public places revealed a dramatic difference. The maximum number observed in the public samples was 270 CFU, while the minimum count was 98 CFU. On the other hand, the semi-public places showed 34–12 CFU (Figures 3 and 4). Even after disinfection, semi-public places had a lower number of detected organisms, ranging from 1 to 3 CFU, while public samples had 2–8 CFU. These findings indicate the importance of cleaning to protect the public from contact pathogens [40]–[42]. The dramatic reduction in the number of viable organisms after disinfection makes the transmission of any infection very unlikely.

This study was not only concerned with the number of contaminating organisms but also their type. Bacteria were identified based on their morphological and biochemical characteristics and reaction to the Gram stain. Among the 10 detected bacterial species, 6 were Gram-positive, and 4 were Gram-negative (Table 1). The Gram-positive *Bacillus* spp. is a foodborne pathogen that can produce toxins causing diarrhea and vomiting. *Staphylococcus* spp. is the leading cause of skin and soft tissue infections such as abscesses, cellulitis, and furuncles. *Enterococcus* spp. causes urinary tract infections. Bacteremia and pneumonia can be caused by the Gram-positive *Micrococcus* and *Staphylococcus* spp. and the Gram-negative *Salmonella* and *Klebsiella* spp. Meningitis could be caused by the Gram-positive *Streptococcus* and the Gram-negative *Klebsiella* and *Salmonella* spp. Both *Enterococcus* and *Klebsiella* spp. can cause wounds and surgical infections [43]. The Gram-negative *Salmonella*, *Campylobacter* spp., and *E. coli* are among the major bacterial contaminants that cause diarrhea and enteritis. Interestingly, among the three detected bacteria in the semi-public places was *Lactobacillus* spp. *Lactobacilli* are normally present in the human body, mainly in the digestive and female genital tract. They help the host in the digestion of certain foods and play a role in pathogen protection [44],[45]. Their potential role as probiotics has gained the attention of scientists all over the globe [46].

Fungal contaminants in the samples were also explored. While samples obtained from the semi-public locations were free from any fungal contamination, those from public locations were contaminated with four fungal genera: *Aspergillus*, *Mucor*, *Rhizopus*, and *Penicillium* spp. (Table 2). *Aspergillus* spp. is the main cause of aspergillosis; the inhalation of *Mucor* spores by people with weakened immune systems can cause lung or sinus infection. *Rhizopus* spp. can cause sinusitis and pneumonia. *Penicillium* spp. are the main cause of superficial infections such as keratitis and otomycosis. In addition, exposure to *Penicillium* spp. is associated with allergic pulmonary diseases, often occupational, such as various cheese workers' lung diseases [47].

## 5. Conclusions

The current study aimed to shed light on the importance of hygiene measures in the protection of the public from infections. The results obtained demonstrate that multi-user video gamepads contain various types of microorganisms and can be the source of cross-contamination and infections. Restricted places, described as semi-public places exposed to a small number of users belonging to the university, showed fewer microorganisms than those located in public places, with poor cleaning measures and exposed to users of different ages, educational levels, and social backgrounds. The organisms were studied from air samples and video gamepads in the selected places. Samples collected after cleaning with alcohol swab pads containing 70% isopropyl alcohol had a dramatic reduction in the number of microorganisms of approximately 97% in the public spaces and more than 91% in the semi-public places. Based on the findings of the current study, authorities are recommended to implement further hygienic measures in public places. The use of hand sanitizers is also recommended before and after using multi-user objects.

## Use of AI tools declaration

The authors declare they have not used Artificial Intelligence (AI) tools in the creation of this article.
